# Small bowel obstruction in the elderly: a plea for comprehensive acute geriatric care

**DOI:** 10.1186/s13017-018-0208-z

**Published:** 2018-10-20

**Authors:** Ekin Ozturk, Marianne van Iersel, Martijn MWJ Stommel, Yvonne Schoon, Richard RPG ten Broek, Harry van Goor

**Affiliations:** 1 Department of Surgery, Radboud University Medical Center, P.O. Box 9101, 6500 HB Nijmegen, The Netherlands; 2 Department of Geriatrics, Radboud University Medical Center, Nijmegen, The Netherlands; 3 Radboud Institute for Health Sciences, Radboud University Medical Center, Nijmegen, The Netherlands

**Keywords:** Small bowel obstruction, Adhesions, Quality of life, Elderly patients

## Abstract

Small bowel obstruction is one of the most frequent emergencies in general surgery, commonly affecting elderly patients. Morbidity and mortality from small bowel obstruction in elderly is high. Significant progress has been made in the diagnosis and management of bowel obstruction in recent years. But little is known whether this progress has benefitted outcomes in elderly patients, particularly those who are frail or have a malignancy as cause of the obstruction, and when considering quality of life and functioning as outcomes. In this review, we discuss the specific challenges and needs of elderly in diagnosis and treatment of small bowel obstruction. We address quality of life aspects and explore how the concept of geriatric assessment can be utilized to improve decision-making and outcomes for elderly patients with a small bowel obstruction.

## Background

Small bowel obstruction (SBO) is a common emergency diagnosis in elderly patients, which occurrence tends to increase parallel to the increasing number of elderly patients requiring acute medical care and emergency surgery [1, 2]. Approximately 10–12% of patients above 65 years presenting with abdominal pain at the emergency department (ED) is diagnosed with small bowel obstruction [2, 3]. Small bowel obstruction in general is one of the most frequent causes of general emergency surgery. In the UK, small bowel obstruction accounts for 51% of all emergency laparotomies [4]. Adhesiolysis and small bowel resection are two of seven main causes counting for 80% of morbidity and death related to emergency surgery [5]. Any emergency surgery in elderly is associated with high morbidity and mortality compared to elective operations [5–8].

Management of small bowel obstruction has advanced over recent years resulting in improved treatment results for small bowel obstruction in the general population [9]. Computed tomography (CT) has been a step forward in detecting different etiologies of bowel obstruction and of the completeness of the obstruction [10]. Adding water-soluble contrast can accurately predict completeness of obstruction and successful conservative treatment [11]. These diagnostic tools led to a more tailored approach and to a reduction of immediate operations. Today, more than 70% of small bowel obstructions are treated successfully by conservative approach, avoiding the risks of a potentially complicated operation [10, 12, 13]. Also, laparoscopic surgery of small bowel obstruction has been introduced as treatment option potentially reducing postoperative morbidity, although this minimal invasive approach is not suitable for every patient and harbours its own complications [14–16].

It is questionable if elderly patients with small bowel obstruction benefit from the progress in the management in small bowel obstruction because of specific challenges and demands in diagnosis and treatment in this patient population [17]. They represent a diverse group consisting of vital elderly patients who have enough resilience to cope with a ‘second hit’ like surgery compared to frail elderly patients with limited reserve capacity and high risk of complications even without surgery. Elderly patients often present with atypical clinical features, causing a delay in diagnosis and progressed disease at first presentation [18, 19]. Most elderly patients have reduced renal function increasing the risk of contrast nephropathy from contrast-enhanced CT [20]. This risk is higher when fluid intake is diminished as in small bowel obstruction. The limited physiological reserves and frailty contribute to worse outcomes in the elderly patient in terms of functional decline, more complications and prolonged stay in hospital, regardless of treatment [6, 21, 22]. High age and common co-morbidities such as diabetes mellitus have been shown to be independent risk factors for mortality in small bowel obstruction [23]. Little is known about the interference of starvation treatment with necessity to continue medication for chronic disease and pre-existing nutritional deficiencies, which all seem a challenge in managing elderly patients with small bowel obstruction.

Regarding goal settings, frail elderly patients judge quality of life to be more important than prolongation of life, which implies a more balanced decision-making from the start (at the emergency department) taking into account psychosocial aspects of life in addition to clinical aspects. Conceivably, a standard approach based on guidelines may not suffice for appropriate decision-making in diagnosis and treatment of the elderly with small bowel obstruction [10, 24].

In this review, we will discuss the specific challenges and demands elderly patients have in diagnosis and treatment of small bowel obstruction and, if literature data are available, we compare these with younger patients. We will discuss quality of life aspects and explore how the concept of geriatric assessment can be utilized to improve decision-making and outcomes for elderly patients with small bowel obstruction. The results presented may contribute to the development of algorithms and guidelines for management of small bowel obstruction in elderly patients.

## Methods

This is a narrative review with scoping aspects [25]. A formal systematic review and meta-analysis was not feasible due to paucity of studies addressing the relevant topics of small bowel obstruction in elderly patients mentioned above.

### Search strategy

PubMed, the Cochrane database and EMBASE database were searched from inception to January 2018. The search strategy was verified by a senior medical librarian for completeness. PubMed studies were identified using Mesh terms ‘intestinal obstruction’, ‘small intestine’, ‘ileum’, ‘jejunum’ and ‘aged’ and free text ‘small bowel obstruction’, ‘elderly’, ‘old’, ‘eldest’ and ‘geriatric’. A same search strategy was performed in Embase, after which duplicates were identified and deleted. Thereafter, the Cochrane database was searched for reviews concerning small bowel obstruction, resulting in three studies that focused on small bowel obstruction.

**Fig. 1:**
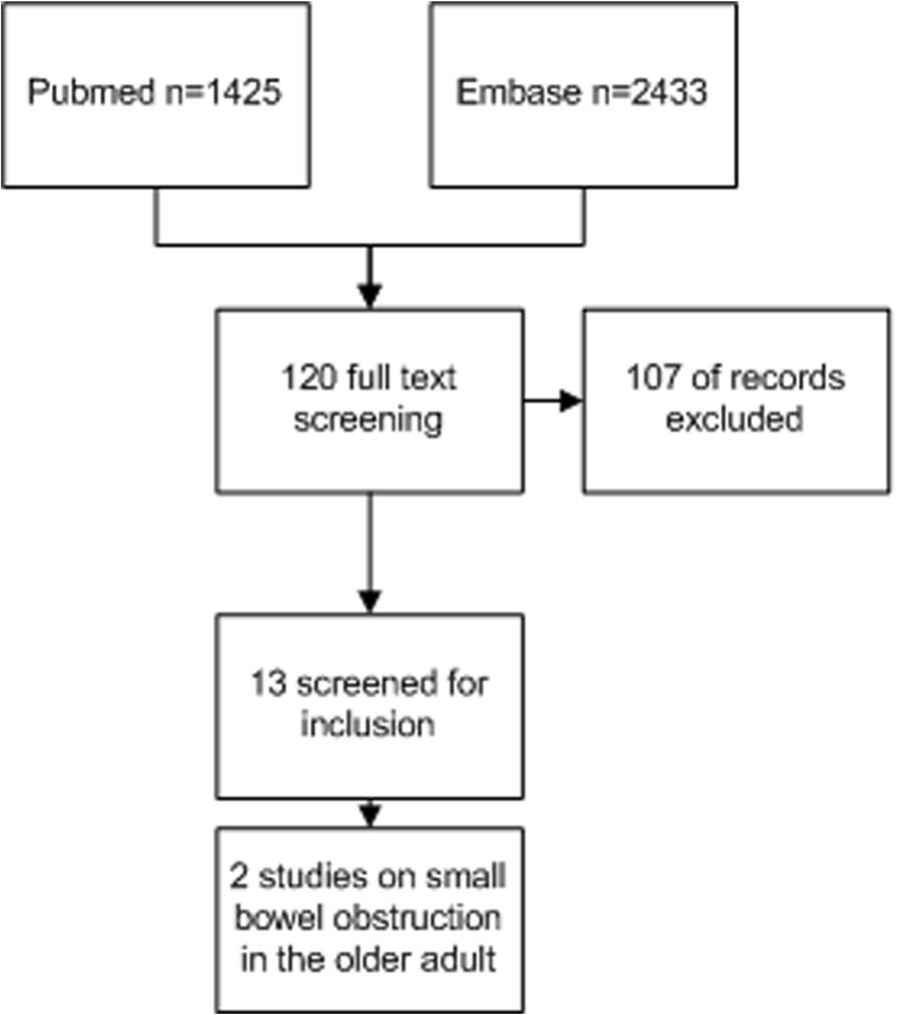
Flow chart of search in PubMed and Embase databases; 120 studies were selected and searched with elderly patients as focus. Exclusion criteria: not focused on older adults, case reports, laparotomy vs laparoscopy, non-English/Dutch/German studies and studies focusing on phytobezoars

One hundred twenty studies from Embase and PubMed were eligible for full-text screening (Fig. 1). Thirteen studies were included for qualitative analysis, whereof two studies were specifically aimed at elderly patients. The Cochrane database search did not reveal a relevant paper. Main reasons for exclusion were as follows: studies did not include elderly patients and studies did not include small bowel obstruction. To ensure our review contained important geriatric themes/considerations that affect diagnosis and treatment of small bowel obstruction, we expanded our search after the systematic search by including more general studies on elderly patients.

This narrative review is divided in two sections; in the first more general section frailty and geriatric assessments are discussed, the second section encompasses the diagnosis and therapy for small bowel obstruction in elderly, decision-making and recommendations for practice or further research. For each section, we summarize and conclude the existing evidence in the literature on small bowel obstruction in the elderly followed by discussing the differences with small bowel obstruction management in the general population.

### Frailty and geriatric assessments

Although different definitions exist, frailty is generally defined as the loss of reserves, after even small insults that lead to increased adverse outcomes [26, 27]. At perioperative screening of elderly patients, frailty is a more important predictor for outcome than age. Frailty not only is independently associated with worse outcomes in terms of morbidity and mortality, but also gives higher risk for functional decline [28]. Around 70 different frailty tests exist, the majority of these tests are aimed at the elective situation, and walking speed is a frequent part of the test. The CSHA Clinical frailty scale by Rockwood et al. consists of 9 points to assess frailty and is relatively simple and quick to perform [29]. The scale correlates well with 5-year mortality and institutionalisation and can easily be used in the emergency situation.

There is only one study that describes frailty assessment in small bowel obstruction [30]. Fifty-three percent of patients in this observational study were mildly to severely frail and 25% were pre-frail, which indicates a high risk for progression to frailty. Unfortunately, frailty was not correlated to outcome, despite existing evidence that frailty negatively affects outcomes in patients undergoing emergency surgery [21].

Patients aged above 70 years with acute abdominal pain (and proven small bowel obstruction) could benefit from screening on frailty [21]. It seems that non-frail patients above 70 years old can be treated in the same way as other adult patients with small bowel obstruction. In frail patients, the higher risks of adverse outcomes and the patient specific goals in life should be added in the decision-making process.

### Geriatric assessment

When patients are suspected or formally tested as being frail, a comprehensive geriatric assessment (CGA) should be performed prior to taking further steps in diagnosis and treatment. Such assessment demands considerable time and attention from geriatricians, but offers the most comprehensive information on frail patients and can help in choosing the treatment that optimally meets the demands and wishes of elderly. Reduced length of stay and lower percentage of change in discharge destination are reported after a CGA [31]. Although there are no data on a (shortened) CGA in small bowel obstruction patients, we believe that optimal care for frail elderly patients with small bowel obstruction will require a multidisciplinary approach from the start, at the ED. Similar to earlier reports on emergency surgery, management of polypharmacy and interactions between treatment of small bowel obstruction and co-morbidities can benefit from dedicated geriatric care and improve results for this frequent condition with high morbidity [4, 32].

There are several models for providing comprehensive geriatric care including geriatric consultation teams, interdisciplinary pathways, aged care consultation at nursing level and primary admittance to a geriatric ward [33, 34]. The optimal model for emergency care, e.g. small bowel obstruction remains undetermined and might vary with local expertise and resources. By all means, the model chosen should fulfil criteria of rapid availability of geriatric competence at the ED 24 h a day and a predefined multidisciplinary care path. We recently started a pilot at the ED of the Radboud University Medical Center (dually headed by a senior geriatrician and trauma surgeon), studying feasibility and short-term efficacy of a multidisciplinary team diagnosing and treating elderly patients with acute abdominal pain. Such a multidisciplinary team includes an emergency physician and nurse (coordinator), a surgeon, a radiologist and a geriatrician. We call this the ‘Car Hood Deliberation’ comparable with consultation between emergency services at the place of an accident.

### Diagnosis

Most common causes of small bowel obstruction are adhesions, tumours and herniations, which are present in more than 90% of cases, with postoperative adhesions counting for approximately 60% [35–37]. Less frequent etiologies of bowel obstruction differ between older and younger adults in prevalence; Crohn’s disease is rare in elderly patients while gallstone impaction is more frequent [36, 38, 39].

Symptom presentation of small bowel obstruction in elderly patients has not been studied in detail, but elderly patients seem to present later and with less profound symptoms at the ED compared to younger patients, resulting in a higher rate of misdiagnosis [3, 40, 41]. In a large cohort study, 52% of elderly patients presenting at the ED with abdominal pain had an incorrect initial diagnosis, compared to 45% of younger patients [40]. Medical staff should especially be aware that elderly patients with acute disease have less pronounced pain, leukocytosis and fever, signs that are relevant discriminating between uncomplicated and complicated small bowel obstruction [42]. There is an increase in pain threshold through lifespan, dependent of type of pain stimulus [43, 44]. Elderly have lower baseline temperatures and in 20–30% of cases show no fever at all in cases of serious infection [45]. Parker et al. studied the usefulness of leukocyte count and other laboratory screening test to distinguish elderly patients in need for acute care surgery from elderly patients with non-surgical emergencies. He found no difference in laboratory values, and 13% of patients in need for acute care surgery had complete normal test results [46]. If applicable to patients with small bowel obstruction, it might be more difficult to recognize strangulation or ischemic bowel in the elderly patient based on physical examination and laboratory screening.

The need for an oral and intravenous contrast-enhanced CT may be omitted in younger patients with high suspicion of adhesions as cause [10]. However, in elderly patients, it is a valuable diagnostic tool to identify patients with small bowel obstruction who require immediate surgery. A study in octogenarians and nonagenarians reported a significant change in small bowel obstruction diagnosis before and after CT [47]. A CT sensitivity and specificity between 90 and 98% for complete bowel obstruction was found in the elderly population [48, 49]. Millet et al. showed significant improvement in diagnosis and management after the use of unenhanced CT for acute abdominal pain in elderly patients [50]. Besides its value of facilitating diagnosis in older patients, unenhanced CT does not harbour the risk of contrast-induced nephropathy. Moreover, other causes or signs can be brought to light, such as unsuspected intra-abdominal neoplasm, intestinal pneumatosis or portal venous gas [47, 50, 51]. When patients have a history of (abdominal) malignancies, a CT may differentiate between malignant bowel obstruction and adhesive obstruction related to previous abdominal surgery [52]. Another, less often utilized imaging tool that is both diagnostic and therapeutic in adhesive small bowel obstruction is water-soluble contrast follow through abdominal film or CT. This has proven to be a reliable diagnostic tool to distinguish complete from incomplete obstruction [53]. Only one randomized study has been performed to assess the therapeutic effects of a water-soluble contrast follow through investigation in combination with somatostatin (analog) in patients with small bowel obstruction older than 65 years. The patients in the intervention group had less surgery, less pain, earlier start of oral intake and earlier stool passage [54, 55].

### Treatment

The cornerstone of non-operative management of small bowel obstruction caused by adhesions is starvation, stomach decompression using a nasogastric tube and fluid resuscitation. This approach seems uniform for younger and older patients. Non-operative management should further include correction of electrolyte disturbances and nutritional support, especially in the frail older patient to avoid delirium, functional decline and complications as a result of starvation and malnutrition [36, 56, 57]. Non-operative management is effective in approximately 70–90% of patients with adhesive small bowel obstruction in general [35, 55, 58]. Though it has a significant failure rate, the nasogastric tube remains relevant in the conservative treatment of small bowel obstruction to initially relieve symptoms and avoid aspiration [10]. Triple-lumen long tubes have been claimed superior to nasogastric tubes in terms of failure [59]. A drawback of the triple-lumen tube is the frequent need for endoscopic placement with use of sedatives, which inherits a risk of pulmonary complications in the frail older patient. Additionally, elderly patients often have hyperactive delirium with a higher risk of accidental tube removal [60].

Starvation treatment can conflict with concurrent treatment of co-morbidities and multiple drug intake [61, 62]. There is surprisingly little evidence to support clinical decision-making on medication during starvation treatment. Clinicians have three options; discontinuing the medication, exempting medication from the starvation regimen, or administering the same or similar drug via other routes, e.g. intravenously. It is generally accepted that medication taken for long-term risk management can safely be discontinued during the course of treatment for small bowel obstruction [63]. Some oral medications can be administered safely with a short period clamping the tube; however, care should be taken in elderly patients with pre-existing dysphagia or neurologic conditions with risk of aspiration of medication. Even though oral ingestion or administering drugs via a tube is often feasible, the uptake of medication is questionable in small bowel obstruction [64]. There is marked paucity in data on complications that might arise from discontinuation or reduced uptake of oral medication in elderly patients with small bowel obstruction. For some medications, alternative routes show different pharmacokinetics with different clinical effects. Well-known examples are beta blockers and benzodiazepines, often prescribed to elderly patients [65, 66].

An ongoing debate in the management of small bowel obstruction is the duration of non-operative treatment that is deemed mandatory to resolve the bowel obstruction before the decision to operate. Most authors apply the 72-h safe-time rule for duration of initial non-operative therapy irrespective of age [10, 12, 55, 67]. It seems, however, that in the elderly, the non-operative treatment is chosen more often from the beginning and that the duration of non-operative treatment is longer compared to the younger population, arguing that the risks of complications and loss of quality of life associated with operation are then avoided [23, 68]. A more conservative approach in the elderly is questionable because the negative effects of delayed surgery seem more pronounced in the elderly patients [36, 55]. In a prospective study, Springer et al. reported a 14% mortality in elderly patients undergoing delayed surgery compared to 3% with early surgery [30]. Causes for increased deaths were not reported. A recent other study showed no difference in outcomes between younger and older patients with small bowel obstruction; however, these results should be interpreted with caution because of the retrospective design and risk selection bias with including the most fit elderly patients [69]. Although it has been recognized that treating elderly patients with small bowel obstruction is somewhat sailing between Scylla and Charybdis, we would make a plea for earlier decision-making for conservative or surgical treatment instead of following the 72-h non-operative treatment time. In this early phase, more comprehensive decision-making should be included in the decision-making process.

### Malignant bowel obstruction

Small bowel obstruction due to malignancy is more common in elderly patients and has a different approach compared to other causes. It has a bad prognosis in the majority of patients, irrespective of age. Survival in general is circa 5 months. This is even lower in patients with gynecological cancers or peritoneal disease, who have a median survival of less than 3 months after surgery [52, 70–72]. Non-operative management shows high failure rates, although palliative treatment with percutaneous decompressing jejunostomy could be considered [73]. The results of palliative surgery in terms of mortality, morbidity and functional decline in the elderly population are not known in detail; however, increased age in general is a known risk factor for morbidity and mortality in malignant small bowel obstruction [71, 74]. In case of peritoneal carcinomatosis, surgery provides little to no relief. It will lead to high morbidity and prolonged hospitalization and very often to non-reparable re-obstruction [72, 75]. Therefore, surgery for malignant small bowel should be abandoned in frail elderly patients keeping in mind quality and not quantity of the remaining short life.

### Quality of life, functioning and decision-making

In elderly patients quality of life considerations and functioning are important in decision-making, particularly regarding surgery. Focus should be laid on goals and wishes in their remaining life [76]. There is little information about the quality of life consequences of surgery for small bowel obstruction in the aged. Recommendations from guidelines on small bowel obstruction hardly help because these are largely based on studies with younger adults [76–78]. Jeppesen et al. surveyed the impact of small bowel obstruction surgery on pain and quality of life and functioning. They found that 19% of patients had pain-related functional impairments after surgery for small bowel obstruction. Patients with chronic postoperative pain had a significantly lower gastrointestinal quality of life score compared with those without postoperative pain (109 (IQR 39) vs. 127 (IQR 19), *P* < 0.001) [23]. Unfortunately, the authors excluded patients older than 75 years in their survey, but considering the relative high risk of complications, it can be assumed that surgery for small bowel obstruction in the elderly seriously impairs quality of life and functioning [6, 30, 79–82]. Scoring systems that predict outcomes in small bowel obstruction and include co-morbidities are currently being validated. These systems however do not include functional state, frailty, or age. Furthermore, they integrate the performed treatment in their score as a predictor of outcome, instead of being a predictor of optimal treatment [83]. Incorporating patient’s preferences in decision-making regarding treatment for small bowel obstruction becomes increasingly important for older patients as they may ‘run out of time’ to overcome the physiological impact of treatment, particularly surgery. Dunn et al. pled for a different approach in old and frail patients with an indication for high-risk surgery. Instead of aiming at a prolonged life, the most relevant question for elderly patients is ‘how he wants to live’ [84]. This certainly applies for patients with a diagnosis of malignant small bowel obstruction.

Decision to refrain from palliative surgery for small bowel obstruction has drawbacks: signs and symptoms of small bowel obstruction remain with often the necessity for the patient to remain in hospital. Still, it would be worth exploring treatment preferences and incorporate them in the palliative approach, particularly in patients with poor quality of life from recurrent obstructions, when an advanced tumour is the cause of obstruction, and in patients with marginal pre-existing general condition.

## Conclusion

There is few evidence regarding diagnosis and treatment of the elderly patient with small bowel obstruction. The scarce literature available demonstrates that elderly patients have an increased risk for complications and mortality and might benefit from earlier surgical intervention. It is important to take patients’ preferences into account starting treatment in general and offering surgery specifically, because an operation may significantly affect quality of life. An assessment of frailty and a comprehensive geriatric approach to the elderly with small bowel obstruction with multidisciplinary specialist care is required from the start and preferably in the ED. More research on management of small bowel obstruction in elderly patients is urgently needed considering the rise in this age group (Table 1).

**Table 1:** Overview of known and unknown facts concerning small bowel obstruction in elderly patients

What this paper adds • Adhesive small bowel obstruction (SBO) accounts for the largest group of SBO. Malignant small bowel obstruction, gall stone ileus and hernia incarceration are more prevalent in elderly patients • Symptom presentation is otypical in elderly patients • CT scan is highly sensitive and specific, and unenhanced CT is a safe method for diagnostics • Frail elderly patients have more morbidity, mortality and functional decline • A comprehensive geriatric approach, including patients’ preference is preferable. • Applied waiting period to surgery might be too long in older patients with SBO	
What is not known? • Functional outcomes of elderly patients after SBO • Optimal ‘safe’ time to wait for resolution of SBO by conservative therapy • Influence of frailty on outcomes in SBO	

**Fig. 2:**
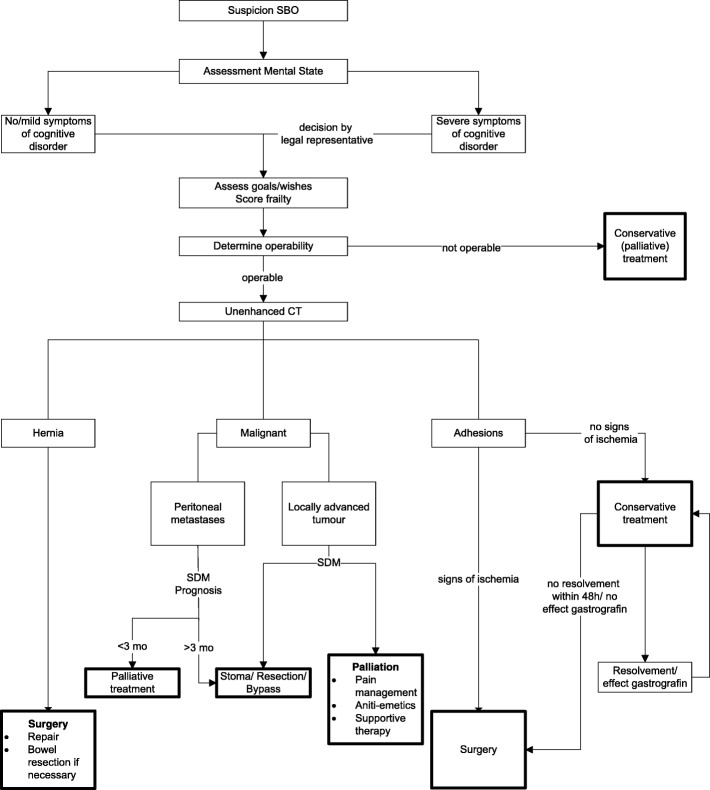
Algorithm with suggested diagnostic and therapeutic steps for the elderly patient with small bowel obstruction. After assessing cognition, frailty and patient goals according to diagnosis, risks and benefits of surgical and conservative management should be weighed. SBO small bowel obstruction, SDM shared decision-making

We conclude that management of small bowel obstruction requires more than just a ‘copy and paste’ of treatment recommendations and guidelines based on studies in younger adults. Based on current knowledge about frail elderly and small bowel obstruction, a flowchart has been developed that offers an approach to diagnosis and treatment of elderly patients with a small bowel obstruction (Fig. 2).

## Abbreviations

CGA: Comprehensive Geriatric Assessment
CT: Computed tomography
ED: Emergency department
SBO: Small bowel obstruction
